# The use of medicinal plants to prevent COVID-19 in Nepal

**DOI:** 10.1186/s13002-021-00449-w

**Published:** 2021-04-08

**Authors:** Dipak Khadka, Man Kumar Dhamala, Feifei Li, Prakash Chandra Aryal, Pappu Rana Magar, Sijar Bhatta, Manju Shree Thakur, Anup Basnet, Dafang Cui, Shi Shi

**Affiliations:** 1 Guangdong Key Laboratory for Innovative Development and Utilization of Forest Plant Germplasm, College of Forestry and Landscape Architecture, South China Agricultural University, Guangzhou, China; 2 Environmental Science Program, Golden Gate International College, Battisputali, Kathmandu, Nepal; 3 Environment Protection and Study Center (ENPROSC), Baneshwor, Kathmandu, Nepal; 4 Central Department of Environmental Science, Tribhuvan University, Kirtipur, Kathmandu, Nepal; 5 State Key Laboratory of Environmental Criteria and Risk Assessment, Chinese Research Academy of Environmental Sciences, Beijing, 100012 P.R. China; 6 Provincial Government Ministry of Social Development, Regional Health Directorate, Dhankuta, Province 1 Nepal; 7 South China Limestone Plants Research Center, College of Forestry and Landscape Architecture, South China Agricultural University, Guangzhou, China

**Keywords:** Corona, COVID-19, Knowledge, Medicinal plants, Pandemic, People, Prevent

## Abstract

**Background:**

Medicinal plants are the fundamental unit of traditional medicine system in Nepal. Nepalese people are rich in traditional medicine especially in folk medicine (ethnomedicine), and this system is gaining much attention after 1995. The use of medicinal plants has increased during the COVID-19 pandemic as a private behavior (not under the control of government). A lot of misinterpretations of the use of medicinal plants to treat or prevent COVID-19 have been spreading throughout Nepal which need to be managed proactively. In this context, a research was needed to document medicinal plants used, their priority of use in society, their cultivation status, and the source of information people follow to use them. This study aimed to document the present status of medicinal plant use and make important suggestion to the concerned authorities.

**Methods:**

This study used a web-based survey to collect primary data related to medicinal plants used during COVID-19. A total of 774 respondents took part in the survey. The study calculated the relative frequencies of citation (RFC) for the recorded medicinal plants. The relationship between plants recorded and different covariates (age, gender education, occupation, living place, and treatment methods) was assessed using Kruskal-Wallis test and Wilcoxon test. The relationship between the information sources people follow and the respondent characteristics was assessed using chi-square test.

**Results:**

The study found that the use of medicinal plants has increased during COVID-19 and most of the respondents recommended medicinal plants to prevent COVID-19. This study recorded a total of 60 plants belonging to 36 families. The leaves of the plants were the most frequently used. The *Zingiber officinale* was the most cited species with the frequency of citation 0.398. Most of the people (45.61%) were getting medicinal plants from their home garden. The medicinal plants recorded were significantly associated with the education level, location of home, primary treatment mode, gender, and age class. The information source of plants was significantly associated with the education, gender, method of treatment, occupation, living with family, and location of home during the lockdown caused by COVID-19.

**Conclusions:**

People were using more medicinal plants during COVID-19 claiming that they can prevent or cure COVID-19. This should be taken seriously by concerned authorities. The authorities should test the validity of these medicinal plants and control the flow of false information spread through research and awareness programs.

## Background

The new coronavirus disease (COVID-19) pandemic has caused global socioeconomic disturbances with a worrisome number of deaths and health issues, and the world has been struggling to find medicine to treat and prevent COVID-19 [1]. A number of combinations and trials have been done, but so far, they have not produced promising results [2–4]. The different types of misinformation related to COVID-19 have been spreading throughout the world through social media [5], including use of medicinal plant products to prevent or cure COVID-19. Due to this situation, ethnobiologists should collaborate with local people and document the medicinal plants used with caution to stop the inaccurate sharing of information [6].

There is a strong inter-relationship between people and plants according to needs [7–10]. People are dependent on plants for different purposes such as for food, medicine, and houses [11–13]. Plant species have always been a fundamental source for the discovery of drugs [14]. People had used medicinal plants to fight against pandemics in the past [15–17], and dependency of people on medicinal plants might have increased in these days around the world as medicinal plants can be an alternative option to prevent COVID-19 [18].

Different researchers have suggested herbal medicine as a potential option to cure or prevent COVID-19 [19, 20]. Countries like China and India are integrating their use with western medicine to boost the immunity power of COVID-19 patients [21, 22]. In China, traditional medicine showed encouraging results in improving symptom management and reducing the deterioration, mortality, and recurrence rates [23]. On the other hand, the World Health Organization (WHO) (2020) claims medicinal plants might be good for the health and in supporting the immune system, but not in preventing or curing COVID-19. The WHO Africa (2020) claims unscientific products to treat COVID-19 can be unsafe for people, as they may abandon self-hygienic practices, may increase self-medication, and may be a risk to patient safety.

Lifestyle, diet, age, sex, medicinal conditions, and environmental factors have been playing an important role in the personal fate towards the severity of COVID-19 [24]. The source of information, such as social media, plays an important role to combat pandemics [25, 26]. People receive information regarding COVID-19 and other diseases from different sources including the social media, local people, national health authorities, and the WHO, based on respondent characteristics such as age and gender as well as occupation, state of their living, and primary mode of disease treatment method [27].

In Nepal, the medicinal plants are often used in the traditional medicine system, which includes Scholarly medical system (The Ayurveda, homeopathy, the Unani, and the Tibetan medicine), Folk medicine (ethnomedicine, community medicine, household medicine, and any other forms of local medicines), and Shamanistic (Dhami-jhankri, Jharphuke, Pundit-Lama-Pujari-Gurau, and Jyotish). Among them, folk medicine system is using more medicinal plants in Nepal [28]. The first scientific research published in ethnobotany is dated back to 1955 [29]. More than 80% of the people in Nepal have been using traditional medicine such as medicinal plants [30, 31]. Medicinal plants are the primary source of healthcare for the people in Nepal and are an integral part of their culture [32, 33]. Most of the people in Nepal have been using medicinal plants as the alternative to allopathic or western medicine [34].

It has also been playing an important role in increasing the economic level of people [35] as Nepal exports medicinal plants to different countries in the world [36]. The elder people living in rural areas have more knowledge of traditional medicine [37].

In Nepal, COVID-19 cases are increasing daily but the health care system is fragile and has a lack of infrastructure [38]. In this context, home remedies, like the use of medicinal plants supported by the relevant authorities, can serve as an alternative option to combat COVID-19. The Nepal government has also valued medicinal plants as an immunity power booster used with prescriptions [39]. But, there a considerable amount of false information spread in Nepal regarding the use of medicinal plants and people are randomly using plants which can go against the traditional methodology and make it difficult to combat COVID-19. The present study has attempted to reveal the status of medicinal plant use in Nepal during COVID-19. Specifically, this study is aimed to address the following objectives: (1) document the status and source of medicinal plants used to prevent COVID-19, (2) know the relationship between the number of plants reported and covariates, and (3) know the relationship between information sources respondents follow and respondent characteristics.

## Methods

### Methods of data collection

A set of questionnaire forms were prepared by Google Form developer. The Google Form was initially tested to validate and understand the response rate from respondents. We followed the code of ethics of the International Society of Ethnobiology [40]. We wrote a consent message to all the people we reached with the form and also placed clearly written consent message at the top of the form. Additionally, we asked a consent question at the beginning of the form for written consent from each respondent. The Google Form was circulated through social media (such as Facebook) and emails in our friend circles asking them to circulate the form with consent message at first as much as possible and inform us whether the form has been sent to others. From our friend circles’ help and our efforts, we reached a total of 998 people throughout the online survey in June 09, 2020, to July 18, 2020, in which a total of 774 (77.55%) people filled the form in different parts of Nepal and provided information about the different variables (Table 1) used for the study.

**Table 1:** Description of the variables used in this study

Variable	Type	Symbol	Categories	Remarks/ Details
Plant number	Numeric	Plants	NA	Number of plant species used
Education	Ordinal	Education	Primary, secondary, university	Formal education of respondents
Occupation	Nominal	Occupation	Agriculture, business, job, jobless, wage earner, remittance	The main source of livelihood of the respondents
Age	Ordinal	Age	> 20 20–29 30–39 40–49 50–59 60–69 70–79	Age of the respondents
Gender	Nominal	Sex	Male (M) Female (F)	Gender of the respondents
Primary treatment mode	Nominal	Primary treatment mode	Allopathy, Ayurvedic, homeopathy	Mode of treatment people normally follow
Source of information	Nominal	Source of information	WHO, national health authorities, social media, local community	Source of information people follow to use medicinal plant
Medicinal plant use	Ordinal	Medicinal plant use status	Increase, decrease, same, never used	The medicinal plant use status during COVID-19 compared to before COVID-19
Recommendation of medicinal plant	Ordinal	Recommendation	Strong, moderate, low, never	Respondents’ recommendation levels were recorded
Living conditions during lockdown	Nominal	Living conditions	Urban, rural	The place of living during lockdown was recorded
Living with family	Nominal	Living with family	Yes No	Respondents living with family or not are recorded
Plant growing conditions	Ordinal	Medicinal plant growing condition	Less, same, more, started, never	Plants’ growing conditions during COVID-19 pandemic
Knowledge about medicinal plant	Ordinal	Knowledge of medicinal plant	Increase, decrease, same, confused	The respondents’ knowledge level on the use of medicinal plant
Habit analysis	Nominal	Habit	Herb, shrub, climber, tree	Types of plant mentioned by the respondents

### Sample population

A total of 774 respondents participated in the survey, of whom 407 (52.58%) were from the urban area and 367 (47.42%) were from the rural area. The age of the respondents varied from 16 to 76 years. Among them, 65.51% were below 30 years of age; all of the respondents were literate, and most of them (69.5%) had attended University. There were more male respondents (60.85%) than female (Table 2).

**Table 2:** Demographic profile of respondents

Demographic parameter	Description	Total respondents (*n* = 774)	Frequency (%)
Age	> 20	31	4.01
	20–29	476	61.5
	30–39	121	15.63
	40–49	64	8.27
	50–59	50	6.46
	60–69	23	2.98
	70–79	9	1.16
Sex	Male	471	60.85
	Female	303	39.15
Education	Primary	36	4.65
	Secondary	200	25.84
	University	538	69.5

### Data analysis

The status of medicinal plants used during COVID-19 (increase, decrease, same, and never used) and recommendation of medicinal plants (strong, moderate, low, and never) was calculated and shown in the bar graph using Microsoft Excel 2013.

The medicinal plants recorded were tabulated in the table with respective scientific, local, and English names with their family and parts (root, stem, leaves, rhizome, roots) used. The scientific names from local name identification followed the Dictionary of Nepalese plant nam e[41] and ethnomedicine study from Nepal [42], and the family assignation in this paper followed the TROPICOS [43]. Finally, we reaffirmed plant species by taxonomic experts from Tribhuvan University Nepal and collected herbarium specimens were deposited in the National Herbarium and Plant Laboratories (KATH) Godawari, Lalitpur Nepal, and specimen codes were presented in a table for each species. For all the species, frequency of citation (FC) and relative frequency of citation (RFC) were calculated following Tardio and Pardo-de-Santayana (2008) [44].

$$ RFC=\frac{FC}{N} $$

where FC = number of respondents who mentioned the use of species and *N* = total number of respondents took part in a survey.

The results of the RFC and the top 10 medicinal plants used are presented in the radar diagram using Microsoft Excel 2013.

The Shapiro test, Kruskal-Wallis test, Wilcoxon test, chi-square test, and related diagrams were drawn using R [45]. The Shapiro test was performed to test the normality of the data. As the data of plant number was not normally distributed, the Kruskal-Wallis test was performed to test the relationship between several plants with an occupation, education level, primary treatment mode, and age class. The Wilcoxon test was performed to see the differences in number of plants reported with gender and place of living during COVID-19 pandemic.

The relationship between information sources and respondent characteristics was shown in the graph and statistically analyzed using the chi-square test.

## Results

### Status of medicinal plant use

**Fig. 1:**
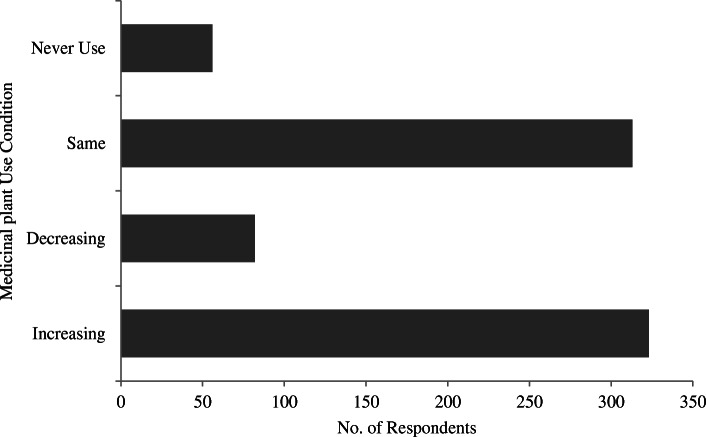
Trend of medicinal plant use during COVID-19

Out of 774 respondents, 323 (42%) respondents agreed that the use of the medicinal plant has increased during COVID-19, whereas 313 (40.44%) agreed the use of medicinal plants during COVID-19 is the same as that of normal condition (Fig. 1).

**Fig. 2:**
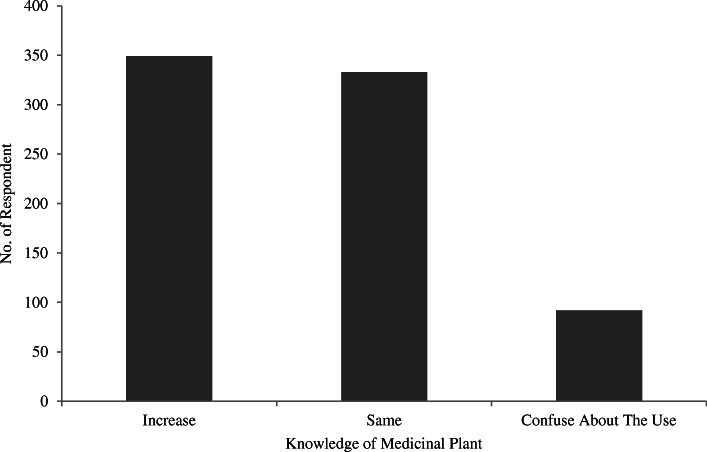
The knowledge level of people on the use of medicinal plants during COVID-19

Most of the respondents, 349 (45.09%), believed that information/knowledge of medicinal plants has increased during COVID-19, 333 (43.02%) believed it is the same as usual, and 93 (11.89%) considered that they are confused about the use of medicinal plants (Fig. 2).

**Fig. 3:**
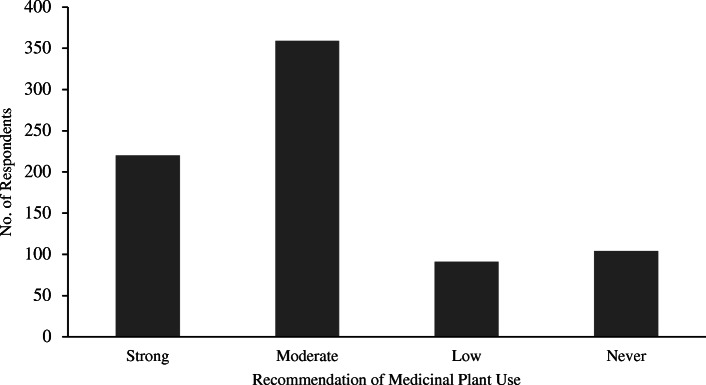
Recommendation of a medicinal plant to prevent and cure COVID-19

A total of 670 (86.5%) of the respondents had recommended medicinal plants to prevent COVID-19, whereas 104 (13.4%) had not recommended. Most of them had made a moderate recommendation (Fig. 3).

### Medicinal plants recorded

**Fig. 4:**
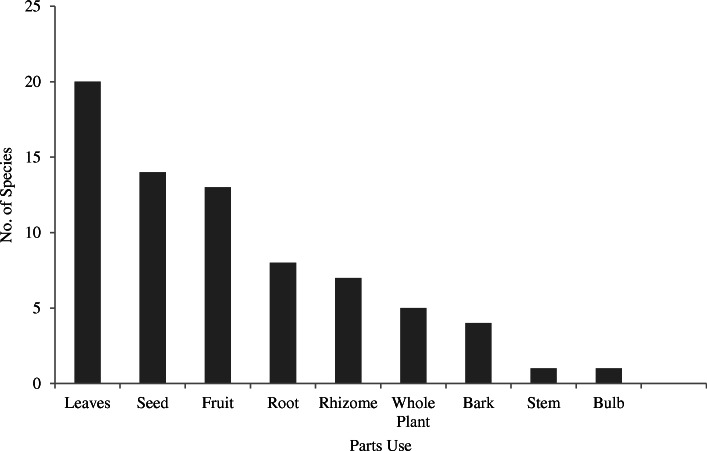
Parts of plants used for medicinal purpose to prevent COVID-19

A total of 60 species of medicinal plants from 36 families and 54 genera were documented as being perceived. Among them, the most common families were Apiaceae (6 species), Zingiberaceae (4 species), Amaryllidaceae (4 species) and Lamiaceae (4species). And most common genus were Allium (3 species), Terminalia (2 species), Mentha (2 species), Cinnamomum (2 species), and Syzygium. Likewise, the most perceived species was *Zingiber officinale* (39.79%) followed by *Curcuma angustifolia* (34.11%). The habit analysis showed that the medicinal plants belonging to herb, shrub, climber, and tree species were 56.67%, 11.67 %, 6.67%, and 25% respectively (Table 3). Leaves (33.68%) were the most predominantly used parts, followed by seeds (23.33%), fruits (21.67%), roots (13.33%), rhizomes (11.67%), whole plant (8.33%), bark (6.67%) stem (1.67%), and bulb (1.67%) (Fig. 4). The most commonly used method of preparations was to grind the parts, boil with hot water or milk, and drink.

**Table 3:** Medicinal plants recorded with scientific name, habit, parts used, mode of use, frequency of citations (FC), and relative frequency of citation (RFC)

Family	Scientific name	English name	Local name	Habit	Parts used	Mode of use	FC	RFC	Herberium specimen code
Acanthaceae	*Justicia adhatoda* L.	Malabar nut	Asuro	Shrub	Leaves	Raw, powder	11	0.014	KATH-01
Amaryllidaceae	*Allium cepa* L.	Onion	Pyaj	Herb	Rhizome	Raw, boil with water	20	0.026	KATH-02
Amaryllidaceae	*Allium hypsistum* Stearn	Nepali aromatic leaf garlic	Jimbu	Herb	Leaves	Powder	1	0.001	KATH-03
Amaryllidaceae	*Allium sativum* L.	Garlic	Lasun	Herb	Bulb	Dried, boil with water	217	0.280	KATH-04
Amaryllidaceae	*Crinum latifolium* L.	Milk and wine lily	Sudarsana	Herb	Root, leaves	Dry powder	3	0.004	KATH-05
Apiaceae	*Carum carvi* L.	Caraway	Kalo jira	Herb	Seed	Raw	2	0.003	KATH-06
Apiaceae	*Centella asiatica* (L.) Urb.	Water pennywort	Ghod tapre	Herb	Rhizome	Raw	3	0.004	KATH-07
Apiaceae	*Coriandrum sativum* L.	Coriander	Dhaniya	Herb	Seed, leaves	Boil with water, powder	7	0.009	KATH-08
Apiaceae	*Cuminum cyminum* L.	Cumin	Jira	Herb	Seed	Raw	13	0.017	KATH-09
Apiaceae	*Foeniculum vulgare* Mill.	Foeniculum fennel	Madhesi souf	Herb	Root, seed	Raw, boil with water, powder	3	0.004	KATH-10
Apiaceae	*Trachyspermum ammi* (L.) Sprague	Ajowan lovage	Jawano	Herb	Seed	Dry powder, boil with water	17	0.022	KATH-11
Asteraceae	*Artemisia indica* Wild.	Mugwort/Indianworm/wood fleabane	Titepati	Herb	Leaves	Powder	1	0.001	KATH-12
Araceae	*Acorus calamus* L.	Sweet flag	Bojho	Herb	Rhizome	Raw	17	0.022	KATH-13
Asphodelaceae	*Aloe vera* (L.) Burm. f.	Indian aloe	Ghiu kumari	Herb	Whole plant	Raw paste with water	15	0.019	KATH-14
Cannabaceae	*Cannabis sativa* L.	True hemp/Indian hemp/marihuana	Ganja	Herb	Leaves	Raw, powder, boil with water	5	0.006	KATH-15
Caricaceae	*Carica papaya* L.	Papaya	Meva	Shrub	Fruit	Powder drink with water or milk, dry, boil with water	1	0.001	KATH-16
Combretaceae	*Terminalia bellirica* (Gaertn.) Roxb.	Bastard myrobalan	Barro	Tree	Fruit	powder	5	0.006	KATH-17
Combretaceae.	*Terminalia chebula* Retz.	Chebulie myrobalan/yellow myrobalan	Harro	Tree	Fruit, bark	Powder, boil with water	18	0.023	KATH-18
Euphorbiaceae	*Euphorbia hirta* L.	Snake weed/asthma weed	Dudhi jhar	Herb	Leaves	Dried, soaked	1	0.001	KATH-19
Fabaceae	*Glycyrrhiza glabra* L.	Licorice	Mulethi	Herb	Root, rhizome	Raw paste	1	0.001	KATH-20
Fabaceae	*Trigonella foenum-graecum* L.	Fenugreek leaf	Methi	Herb	Seed, leaves	Raw, fresh, paste	6	0.008	KATH-21
Gentianaceae	*Swertia chirayita* (Roxb. Ex fleming) Karsten	Chiretta	Chiraito	Herb	Whole plant	Raw, paste, powder, boil with water	2	0.003	KATH-22
Lamiaceae	*Mentha arvensis* L.	Pepper mint/field mint	Pudina	Herb	Whole plant	Powder, boil with water, paste	37	0.048	KATH-23
Lamiaceae	*Mentha piperita L.*	Peppermint	Babri	Herb	Seed	Dried, boil powder with water or milk,	2	0.003	KATH-24
Lamiaceae	*Ocimum basilicum* L.	Basil	Tulasi	Herb	Leaves, seed	Dried, boil with water or milk	142	0.183	KATH-25
Lamiaceae	*Salvia rosmarinus* Spenn.	Rosemary	Dauni	Herb	Flower	Boil, paste	2	0.003	KATH-26
Lauraceae	*Cinnamomum zeylanicum* Breyn.	Cinnamon bark	Dalchini	Tree	Bark	Boil with water, powder	23	0.030	KATH-27
Lauraceae	*Cinnamomum tamala* (Buch.-Ham.) T. Ness & Eberm	Cinnamon leaf	Tej pat	Tree	Leaves	Paste, raw	1	0.001	KATH-28
Marantaceae	*Maranta dichotoma* (Roxb.) Wall.	Cool mat	Shital pati	Herb	Leaves	Dried, raw	4	0.005	KATH-29
Melanthiaceae	*Paris polyphylla* Sm.	Love apple	Satuwa	Herb	Rhizome	Powder, paste	1	0.001	KATH-30
Meliaceae	*Azadirachta indica* A. Juss.	Neem tree	Nim	Tree	Leaves, bark	Boil with water, dried	73	0.094	KATH-31
Menispermaceae	*Tinospora cordifolia* (Willd.) Miers.	Gulancha tinospara	Gurjo	Climber	Stem	Boil with water or milk	74	0.096	KATH-32
Moraceae	*Ficus religiosa* L.	Peepal tree	Pipal	Tree	Leaves	Raw	2	0.003	KATH-33
Myrtaceae	*Syzygium aromaticum* (L.) Merr.& L.M. Perry	Clove	Lwang	Tree	Flower	Raw, paste	12	0.016	KATH-34
Myrtaceae	*Syzygium cumini* (L.) Skeels	Java plum	Jamun	Tree	Fruit, leaves	Raw	2	0.003	KATH-35
Myrtaceae	*Psidium guajava* L.	Guava	Amba	Tree	Leaves	Powder boil with water or milk	3	0.004	KATH-36
Myristicaceae	*Myristica fragrans* Houtt.	Nutmegs	Jayaphal	Tree	Seed	Raw	2	0.003	KATH-37
Oleaceae	*Nyctanthes arbor-tristis* L.	Night jasmine/coral jasmine	Parijaat	Tree	Leaves	Paste	4	0.005	KATH-38
Orchidaceae	*Dactylorhiza hatagirea* (D. Don) Soó	Orchid	Panc aunle	Herb	Tuber, root	Powder, paste	2	0.003	KATH-39
Oxalidaceae	*Averrhoa carambola* L.	Star fruit	Kantara	Tree	Fruit	Powder, boil with water or milk	1	0.001	KATH-40
Oxalidaceae	*Oxalis corymbosa DC.*	Pink wood sorrel	Cariamilo	Herb	Leaves	Raw	1	0.001	KATH-41
Pedaliaceae	*Sesamum indicum* L.	Sesame	Til	Herb	Seed	Raw, juice	1	0.001	KATH-42
Piperaceae	*Piper nigrum* L.	Black pepper	Marich	Climber	Fruit	Boil with water	15	0.019	KATH-43
Phyllanthaceae	*Phyllanthus emblica* L.	Emblic myrobalan	Amala	Tree	Fruit	Paste, soaked	23	0.030	KATH-44
Plantaginaceae	*Bacopa monnieri* (L.) Edwall (L.) Wettst.	Thyme leaved graticula	Brahmi	Climber	Whole plant	Raw, paste, dried, soaked	1	0.001	KATH-45
Poaceae	*Cymbopogon* citrates (DC.) Stap.f.	Lemon grass	Pirhe ghans	Herb	Whole plant	Raw boil with water	4	0.005	KATH-46
Ranunculaceae	*Delphinium denudatum* Wall. ex Hook. f. & Thomson	Jadwar	Nirbisi	Herb	Root	Dried,boil with water	1	0.001	KATH-47
Rosaceae	*Potentilla fulgens* Wall. Ex Hook.	Himalayan cinquefoil	Bajradanti	Herb	Root	Raw	1	0.001	KATH-48
Rosaceae	*Rosa alba* L.	Rose	Gulaph	Shrub	Petals	Raw, dried	2	0.003	KATH-49
Rutaceae	*Aegle marmelos* (L.) Corr.	Bael fruit	Bel	Tree	Leaves, bark, root, fruit, seed	Boil with water	1	0.001	KATH-50
Rutaceae	*Citrus aurantifolia* (Christ.) Swingle	Lime/lemon	Kagati	Tree	Fruit	Raw, juice, boil with water	116	0.150	KATH-51
Rutaceae	*Zanthoxylum armatum* DC.	Nepal pepper/prickly ash	Timur	Shrub	Fruit	Raw	13	0.017	KATH-52
Solanaceae	*Capsicum annuum* L.	Capsicum chilly	Khursani	Shrub	Fruit	Raw mixed with vegetable	2	0.003	KATH-53
Solanaceae	*Withania somnifera* (L*.*) Dunal	Winter cherry	Ashvagandha	Shrub	Root, seed, leaves	Boil with water, powder, paste	1	0.001	KATH-54
Theaceae	*Camellia sinensis* (L.) Kuntze	Tea	Chiya	Shrub	Leaves	Paste, raw boil with water	2	0.003	KATH-55
Vitaceae	*Vitis vinifera* L*.*	Vine grape	Dakh	Climber	Fruits	Raw	1	0.001	KATH-56
Zingiberaceae	*Curcuma angustifolia* Roxb.	Turmeric	Besar/Haledo	Herb	Rhizome	Boil with water or milk, raw, powder taken with water or milk	264	0.341	KATH-57
Zingiberaceae	*Amomum aromaticum* Roxb.	Black cardamom/Nepal cardamon	Alainchi	Herb	Fruits	Boil with water or milk, powder boil with water or milk	4	0.005	KATH-58
Zingiberaceae	*Elettaria cardamomum* (L.) Maton	Cardamon fruit	Sukumel	Herb	Seed	Boil with water, powder taken with water or milk	1	0.001	KATH-59
Zingiberaceae	*Zingiber officinale* Rosc.	Ginger	Aduwa	Herb	Rhizome	Boil with water, paste, powder	308	0.398	KATH-60

### Relative frequency of citation

**Fig. 5:**
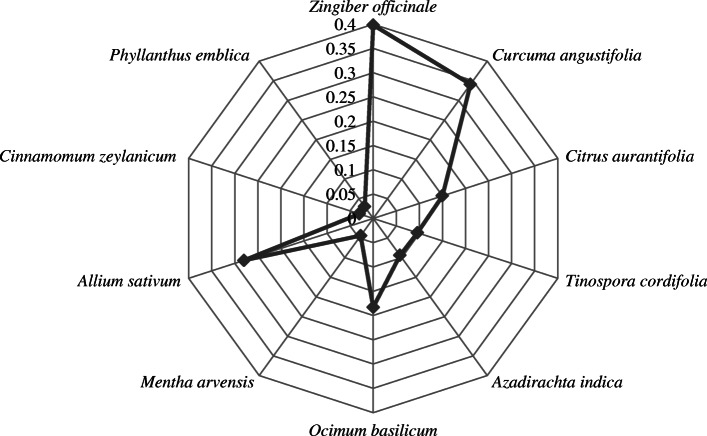
List of top ten ranked plant species reported by respondents shown the frequency of citation

The relative frequencies of citations ranged from 0.001 to 0.398 and for ten most cited species value ranged from 0.03 to 0.398. The most cited species was *Zingiber officinale* (308 times cited and frequency of citation was 0.398) followed by *Curcuma angustifolia* (264 times cited and frequency of citation was 0.341) (Fig. 5).

### Source and cultivating conditions of medicinal plants

**Fig. 6:**
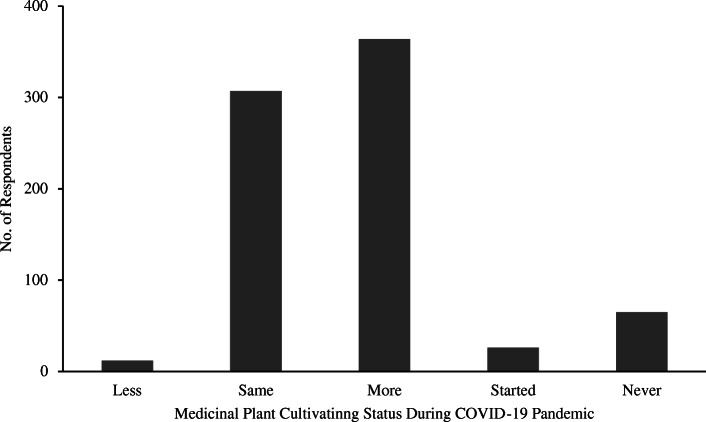
The medicinal plant cultivation status during COVID-19

The respondents had mentioned that they were getting medicinal plants from home gardens (45.61%), markets (32.03%), and jungles (10.73%), and the remaining respondents were getting medicinal plants from all of the above three sources. Most of the respondents were also cultivating (47%) more medicinal plants during COVID-19 than before, and few have just started (3%) (Fig. 6).

### Number of plants reported and covariates

**Fig. 7:**
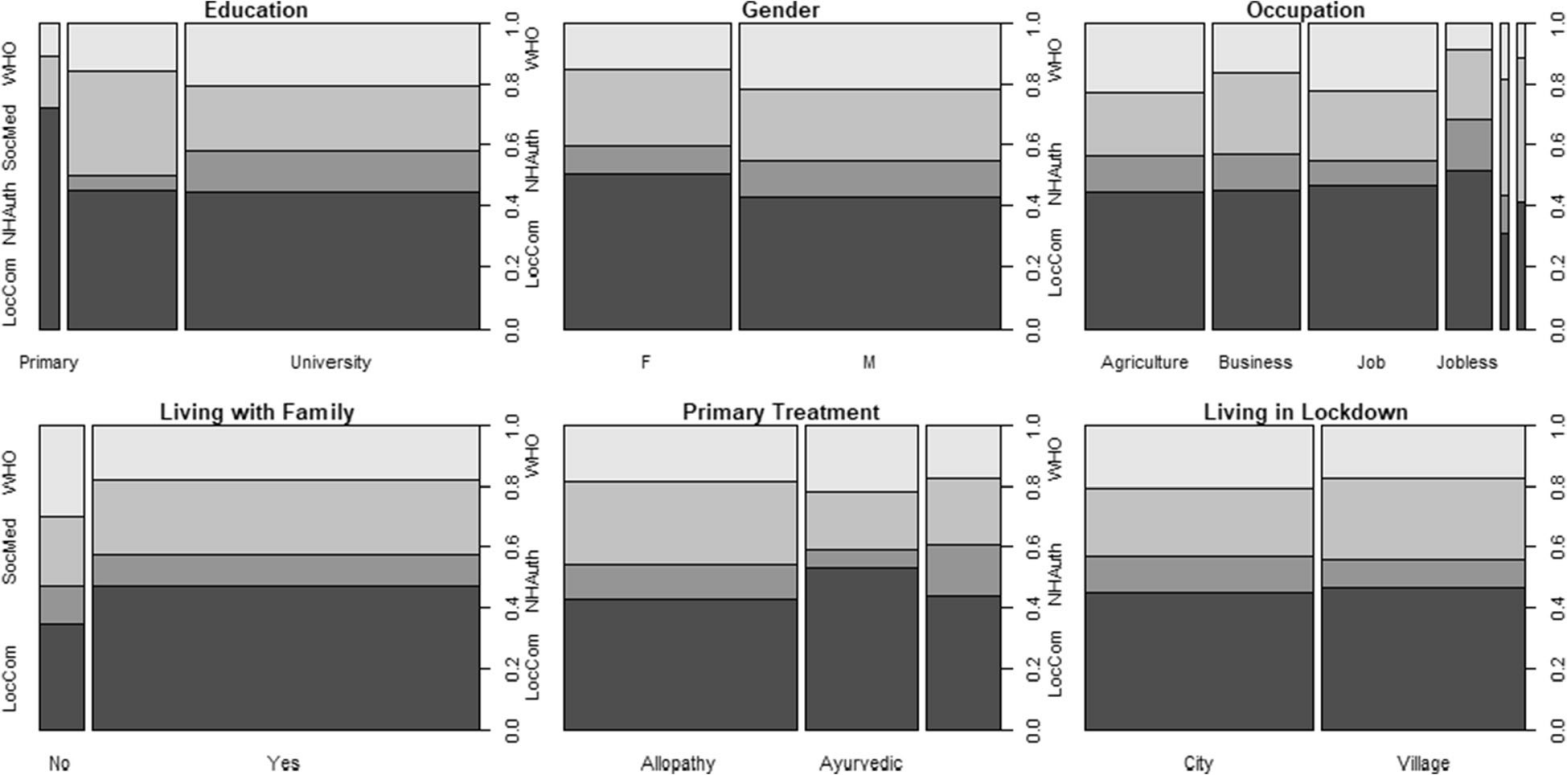
Graphical representation of plant use as a preventive method against COVID-19 by respondents

The number of reported plants used by individual respondents ranged from 0 to 12 (Fig. 7). In the occupational category, people who were engaged in agriculture and those with jobs used comparatively more medicinal plants than others, but the difference was not significant (Kruskal-Wallis, *χ*^2^= 7.921, df = 5, *p* = 0.1606). The people with university-level education were using more plant species compared to people with secondary-level and primary-level education, and the differences were statistically significant ( Kruskal-Wallis, *χ*^2^ = 50.736, df = 2, *p* = < 0.0001 ). The people living in the city were using more plants than people living in the village, which was statistically significant (*W* = 85818, *p* = 0.0002). The people whose primary method of treatment was allopathic were using a statistically significant low number of plants (Kruskal-Wallis, *χ*^2^ = 32.524, df = 3, *p* = 0.0001) compared to the respondents whose primary methods of treatment were Ayurvedic and homeopathic. The female respondents were using more plants than males; the difference in the use of plants by males and females was statistically significant (*W* = 77489, *p* = 0.03864). Age group of 20–29 and below (< 20) reported more number of species being used. The number of medicinal plant species reported was statistically significantly different among the age groups (Kruskal-Wallis, *χ*^2^ = 25.484, df = 6, *p* = 0.0003).

### Information sources

**Fig. 8:**
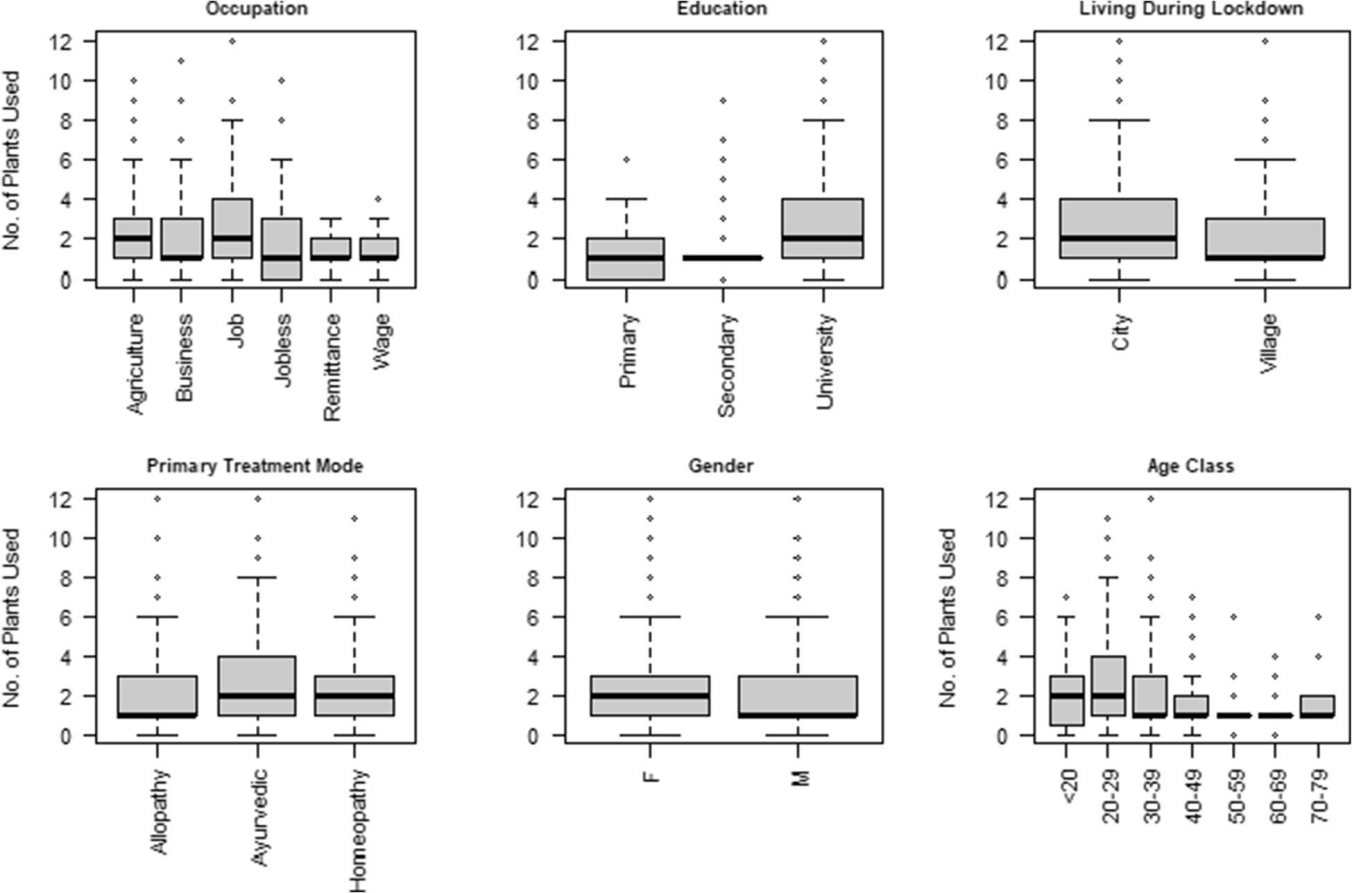
Graphical representation of information sources with respondent characteristics

People are using different sources to prevent COVID-19, such as social media like Facebook Twitter, official information from the World Health Organization, the national health authorities, and local communities (Fig. 8). The information adopted from social media is risky but in significant proportion, more than 25% of secondary education respondents and female respondents are using social media information, and there was a statistically significant relationship between information source and gender (*χ*^2^ = 8.0304, *p* = 0.0459). The relationship between information source and education was statistically significant (*χ*^2^ = 34.714, *p* = 0.0005). The jobless people were following the local community for obtaining information (more than 50%), and the relationship between the source of information and occupation was marginally significant (χ^2^ = 23.863, *p* = 0.0699). The people living with their families were depending more on local communities and social media for plant use information (more than 50% and 25% respectively), and the relationship between the source of information and living with the family was statistically significant (*χ*^2^ = 7.9621, *p* = 0.0445). The people who using Ayurvedic as the primary treatment were mainly following information provided by the communities (more than 50%), and there was a statistically significant association between the information source and the primary treatment method (*χ*^2^ = 17.406, *p* = 0.0095). The people living in the city and village during the lockdown of COVID-19 both followed similar sources of information, and there is no significant association between source of information and people living in lockdown (*χ*^2^ = 4.6375, *p* = 0.2054).

## Discussion

### Status and sources of medicinal plant

Medicinal plants have attracted the attention of several stakeholders around the world [46]. They have chemical diversity and can play a significant role in new drug development [47]. In this study, the majority of respondents in Nepal reported that the use of medicinal plants has increased during COVID-19 and also believed that information about the medicinal plants has increased, and most of them recommend medicinal plants to prevent COVID-19. Researchers such as Rastogi et al. (2020) and Vellingiri et al. (2020) have claimed that medicinal plant-based treatments should be beneficial to treat and prevent COVID-19 [20, 48]. Yang et al. [49] reported that plant species traditionally used as food can help to enhance the immune system of the body and help to prevent the manifestation of COVID-19 [50]. In the past, medicinal plants were combined with western medicine to treat a similar disease, severe acute respiratory syndrome (SARS) [51].

There is no effective medicine available so far for the treatment of COVID-19; medicinal plants are being used globally that might have increased the demand for medicinal plants [52]. Some plants are useful to treat viral disease, but COVID-19 is a new disease, and the effectiveness of the medicinal plants to cure it has not been tested yet. Therefore, the excessive use of medicinal plants, however, could be problematic and is a matter of concern. Easy access to social media which often publish unreliable advertisements might have a role to play in the increasing use of medicinal plants. Moreover, local availability of medicinal plants and an incorrect belief that medicinal plants have no side effects among people might also be responsible for the same. All the stakeholders including ethnobotanists and community leaders should come together to educate people about the proper use of medicinal plants.

### Medicinal plants recorded and frequency of citation

We recorded a total of 60 plant species, and most of the species were similar to the study based on a preliminary survey in five heavily affected cities, Wuhan, Milan, Madrid, New York, and Rio de Janeiro, and twelve less-affected rural areas, Appalachia, Jamaica, Bolivia, Romania, Belarus, Lithuania, Poland, Georgia, Turkey, Pakistan, Cambodia, and South Africa, which recorded 193 plant taxa from 69 families [53]. A study in Morocco had recorded a total of 23 species which include some similar species viz. *Allium sativum*, *Allium cepa*, and *Zingiber officinale* [54]. A study from India recorded 15 species [55]. A study from China have screened 26 medicinal plants for possible treatment of COVID-19 [56]; likewise, other studies from China have discussed about medicinal plants similar to our study [57]. A study from Bangladesh screened 149 plants from 71 families and found they have potential molecules for preparing a drug for the treatment of COVID-19 [58].

Most of the species reported in this study are locally available, home garden species, and used for daily food at home. The leaves were the most used parts of the plants corroborating the findings of other related studies in Asia [59, 60]. The use of leaves is mainly due to the presence of active secondary metabolites [61]. Underground parts, such as roots and rhizomes, are rich in bioactive constituents [62, 63]. However, indiscriminate use of underground parts might lead to conservation threats particularly to wild species [64]. Similarly, the use of bark in an excessive amount and the whole plant use might create problems in conservatio n[65].

The citation of species might have been influenced from social media along with the cultural, religious, and community leaders within Nepal and neighboring India. For instance, the famous Hindu Swami Ramdev of India has suggested that *Tinospora cordifolia* boiled in water, *Curcuma angustifolia*, *Zanthoxylum armatum* powder, and *Ocimum tenuiflorum* leaves can prevent COVID-19 (written in India TV News of 14 March 2020). The most cited species in this study are also the most commonly used species in Nepal, such as *Zingiber officinale*, *C. angustifolia*, and *Allium sativum*. These species are planted in almost every household of rural Nepal, and these species are also listed by the Nepal Ministry of Health & Population Department of Ayurveda & Alternative Medicine, Teku, Kathmandu, as an alternative medicine to boost the immunity power of people [66]. Plants like *Curcuma angustifolia*, *Cuminum cyminum*, *Allium sativum*, *Terminalia bellirica*, *Z. officinale*, *O. tenuiflorum*, *Cinnamomum* species, *Piper nigrum*, *Vitis vinifera*, and *Citrus spp.* were also recommended by the Indian Government to boost immunity power but does not claim to cure or treat COVID-19 [67]. Some of these medicinal plants used might show a placebo effect on people as treatment of diseases like COVID-19 depending on multiple factors such as psychological factor [68].

The medicinal plants reported in the study have different chemical compounds and constituents that have been proved in treating different diseases and ailments. *T. bellirica, Cinnamomum species*, *Piper nigrum*, dry *Z. officinale*, and raisin contain phytonutrients, chlorophyll, vitamins, minerals, eugenol, and a bioactive compound; *Z. officinale* contains sesquiterpenes [69].

Chemical constituents 8-Gingerol and 10-Gingerol from *Z. officinale* were active against COVID-19 [70]. COVID-19 patients might have a cytokine storm [71, 72], and *Curcuma* species like *angustifolia* and *caesia* have the capacity to block cytokine release [73]. *Allium sativum* contains sulfoxide, proteins, and polyphenols like bioactive sulfur-containing compounds which are antiviral with immunostimulatory potential [74, 75]. *Tinospora cordifolia* has alkaloids, glycosides, lactones, and steroids with immunomodulatory roles and can treat fever, chronic diarrhea, and asthma [76, 77]. Citrus species contain polysaccharides and polyphenolic compounds which improve the immunity of body [78]. *Ocimum* species like *Ocimum tenuiflorum* extract contains Tulsinol (A, B, C, D, E, F, G) and dihydrodieuginol that possess immunomodulatory and Angiotensin-converting enzyme 2 (ACE II) blocking properties to inhibit replication of coronavirus [79]. *Phyllanthus emblica* is antioxidative and anti-inflammatory, and its extract Phyllaemblicin G7 has the potential to treat COVID-19 [80]. *Azardirachta indica* extracts Nimbolin *A*, Nimocin, and Cycloartanols (24-Methylenecycloartanol and 24-Methylenecycloartan-3-one) have shown potential to inhibit COVID-19 [81]. *Mentha arvensis* possess eugenol, terpenes, and flavonoids which are good antioxidants and modulators of xenobiotic enzymes which help to inhibit COVID-19 [82]. *Cinnamom species* like *Cinnamom unverum* contains antioxidant and antiviral compounds (eugenol, cinnamic acid, caryophyllene) which might help to inhibit COVID-19 [83].

The species with a lower frequency of citation are also useful in some way; *Camellia sinensis* has immunomodulatory properties due to the presence of epigallocatechin gallate, quercetin, and gallic acid in its leaves [84]. *Euphorbia* species like E*uphorbia thymifolia* has antioxidant and antiviral activities [85]. Functional food such as *Allium cepa*, *Nigella sativa*, *Carica papayas*, and other species are functional food; they possess immunomodulatory properties in several ways and help in effective health management if taken in an adequate manner [50]*.* However, there is no proper research and scientific evidence supporting that medicinal plants can prevent or cure COVID-19. The use of medicinal plants is traditional and has a long history with its own theory, like traditional Chinese medicines whose composition is typical and complicated. A creative evaluation system should be developed before its use to prevent or treat COVID-19 [86]. Some researchers have suggested natural products obtained from plants might be an alternative option to treat COVID-19 [87, 88].

But at present, the use of different, unproven medicine, as well as herbal medicine, has been the only way to protect vulnerable patients and such medicines should not be overlooked, or taken without the prescription from a health personnel [50]. The effectiveness of above-mentioned medicinal plants should be tested scientifically then added to the discovery of drugs used to treat COVID-19.

### Source and cultivating conditions of medicinal plants

Most of the respondents obtained medicinal plants from home gardens or farms. It is interesting to find that people are cultivating more medicinal plants during COVID-19, which is a positive sign for the development of gardening or farming practices in the country. This type of activity will support the sustainable conservation of medicinal plants. However, collecting medicinal plants from the jungle will cause several issues in the conservation of plants [89]. Different types of actions can be taken to conserve and for the sustainable use of such species, including assessing the conditions of plant use and their presence as well as policy formation [90]. Some people have also just started to plant medicinal plants which is a good sign for the sustainable livelihood in Nepal.

### Number of plants reported and covariates

The use of medicinal plants depends on several covariates, such as occupation, education level, age, class, living condition, and treatment methods that people usually follow. The sociocultural acceptance of people vary within different places and communities [91]. People living in villages most live with their families in Nepal, and studies have found that the use of medicinal plants usually comes from families [92]. During COVID-19, well-educated people perceived more medicinal plants in Nepal, contrary to the results of other studies, which found that well-educated people often rely on modern medicine for treatment [93]. Females reported more medicinal plants than males, similar to other studies [94], probably because women are more involved in household work and invest more time in the kitchen, caring for their family, and in food and health, as well as in farm work such as cutting grasses and collecting fodder. People adopting agriculture reported a higher number of medicinal plants, which may be because they have easier access to medicinal plants. In Nepal, people with agricultural occupations and living in rural areas used more traditional methods to stay healthy [95]. The job holders also reported comparatively more number of plants.

Interestingly, the youths (age groups below 30) have reported using more medicinal plants, probably because they lived with their families and learned more about the medicinal plants from the elders. This group is also the most active group on social media. Most respondents also claimed that they were more aware of the medicinal plants during COVID-19, which is a good sign as the research by Tiwari et al. (2020) has mentioned that young people are forgetting the use of medicinal plants. However, the misunderstanding of medicinal plants is also dangerous, and the stakeholders need to think about and provide accurate information to the young people [96]. Young people should follow a reliable source to obtain information about medicinal plants. People who primarily use Ayurvedic and homeopathy remedies reported more number of medicinal plants. The use of plants and the acquisition of knowledge usually depends on the culture and primary health care system [97].

### Information sources and respondent characteristics

The source of information is the key to using medicinal plants, and it is not good to follow social websites and rely on them, as the usefulness and accuracy of messages regarding COVID-19 provided by social media such as YouTube have not been tested [98]. However, in this study, a large number of respondents were found to be engaged in social media to obtain information regarding COVID-19. Most of the people were not relying on the WHO and national health authorities, similar to the study of Bhagavathula et al. [99]. Most well-educated people, female, job holders, people living with families, people who are following allopathy as a primary treatment, and people who live in the village are all following social media to obtain knowledge of prevention methods and using medicinal plant-based on the source which might be incorrect and thus harmful. This is because the frequent use of social media and the practices of using several sources of social media have caused an overload and increased people’s concerns [100].

This study recommends the use of official websites of the WHO and national health authorities to gain information regarding COVID-19. Most people also rely on the communities for the use of medicinal plants which might cause traditional malfunction. Therefore, it is unwise to adopt unscientific sources of information and use medicinal plants privately. The correct use of medicinal plants passes from generation to generation, which is usually applicable to old diseases. No valid medicine has been developed to prevent or cure COVID-19 so far. The COVID-19 pandemic has created a large crisis, and it needs large-scale behavior changes [101]. For instance, we need to change our behavior and follow valid information to use different preventive measures to be free from COVID-19. The collaboration between diverse stakeholders such as the government, volunteers, people, and other sectors is deemed necessary to transmit information and respond to crisis through improving information flow [102]. Different studies on herbal remedies are deemed necessary which would be helpful to prepare an antiviral drug against COVID-19 as well as to help prevent going against traditional methodology related to the use of medicinal plants [103]. There is an urgent need to disseminate a high level of public awareness to prevent misinformation regarding treatment and prevention measures of COVID-19 [104].

### Limitation of the study

This is online survey based study. The questionnaire was mostly circulated among the educated social network colleagues of ours as they can read and understand about the issues, provide their consent, and fill the form similar to other studies from the globe. This might create some bias on the study, but during extreme condition (such as COVID-19 lockdown) this is one of the prime ways to get information and help deal with the extreme situation. Researchers have reported that well-educated people preferred to follow modern medicine, but during COVID-19 time educated people were aware about the medicinal plants as opportunistic medicine [105, 106]. This behavior of educated people helps to increase concern of them on medicinal plants. Further, a field-based study might cover responses from all levels and classes of people with quantification of uses.

## Conclusion

This study found that medicinal plants used and the beliefs related to them have increased during COVID-19. A total of 63 medicinal plant species used to prevent COVID-19 were investigated and recorded. The frequently used plants in the home were recorded more in comparison to other plants. The plants’ cultivation status have increased during COVID-19. The use of medicinal plants was associated with social and demographic variables. Likewise, the source of medicinal plants also varied with the demographic social factors of the respondents. This study recommends undertaking studies of medicinal plants used during COVID-19. The validity and reliability of such medicinal plants should be tested further by phytochemical and pharmacological research, and invalid information should be monitored and controlled in different social media platforms and communities. It is recommended that people follow information from authentic sources related to the COVID-19 pandemic.

## Data Availability

All data have already been included in the manuscript. We are willing to share the data generated and analyzed during the current study.
